# Healthy buildings, healthy people

**DOI:** 10.2471/BLT.18.020318

**Published:** 2018-03-01

## Abstract

An Australian medical research centre and a Norwegian hospital have won acclaim for their healthy designs. Sima Barmania reports.

When researcher Melkam Kebede started her five-year fellowship at the Charles Perkins Centre at the University of Sydney, Australia, she was struck by the abundance of natural light and design features that encouraged people to move and mingle. 

“I’ve worked in many places before, but never seen laboratories with so much natural light,” says Kebede, whose research is on the links between type 2 diabetes and obesity. “Usually we are separated by brick walls. Here there are glass walls between the labs and offices.”

The design of the building reflects both the function and philosophy of a health research centre focused on finding new ways to combat noncommunicable diseases, such as heart disease, stroke, diabetes and obesity.

An enormous white curved staircase that feeds into a series of open-plan corridors on each of the six floors is the central feature of the building.

“The stairway keeps us fit. Many people working here have never used the lifts,” Kebede says, adding that the stairway is also the place “where people meet and share ideas.”

“The stairway keeps us fit. Many people working here have never used the lifts.”

Melkam Kebede

The Charles Perkins Centre is named after the first man of Aboriginal descent to graduate from the University of Sydney. The centre opened its doors in 2014 and was shortlisted the following year for the World's Best Building prize in the higher education and research category at the annual World Architecture Festival.

For Stephen Simpson, the director of the centre, it was important to create an environment with many meeting points that “draws people together in a campus-like building experience.”

The centre includes a hospital clinic and brings together experts from a range of disciplines, from philosophy and economics to engineering, maths, metabolic science and medicine.

“The centre houses more than 1000 researchers, many working on multidisciplinary projects, and hosts hundreds of public events each year,” Simpson says.

A dedicated team from the university prepared the brief for architect Richard Francis-Jones, based on input from more than 20 user groups, and they all worked closely together to reflect the centre’s public health values in the building design.

“The atrium, which is the hub of the building, with its white, organic, curved surfaces, creates a beautiful space where people like to spend time,” says Francis-Jones.

The building is designed to increase natural light, access to fresh air and to encourage people to be physically active. “There is a lot of research which supports the benefits of natural light during the day in tune with our circadian rhythms and access to fresh air,” says Francis-Jones.

“An environment that inspires us, results in happier, more engaged staff and, in turn, increases their productivity,” Francis-Jones says.

Akershus University Hospital on the edge of the Norwegian capital of Oslo is another new building that reflects healthy values. It was awarded the title of the world's best health project over 40 000m^2^ by the International Academy for Design and Health in 2015.

The Norwegian hospital is a perfect example of what Alan Dilani, the Swedish academy’s founder and chief executive officer, calls salutogenic design.

During his doctoral research on the planning and design of health facilities in Stockholm, Dilani signed up for courses at the Karolinska Institute of health sciences.

Drawing on the work of sociologist Aaron Antonovsky, who coined the term salutogenesis in the 1970s, referring to the factors that promote health as opposed to the “pathogenic” factors that cause disease, Dilani developed his own theory of salutogenic design.

“Salutogenic design aims to create physical environments that promote health and wellbeing and, ultimately, healthy societies,” Dilani says.

Far from being a niche approach, Dilani argues that “salutogenic design must become the core essence of all architecture, changing the way we design environments where people live, work and play.”

“Complex buildings, such as hospitals and other public institutions, should be designed to reduce stress,” says Dilani, who was inspired by the work of Swedish occupational health expert Lennart Levi, one of the pioneers of psychosocial medicine in the 1970s.

“According to Levi’s work on the effects of stress, our physical environment can promote health or disease,” Dilani says. “That is why emotions and experiences are central parts of the health process and can be strengthened by exposure to positive stimuli from surrounding environments where we live, work and play.”

Design factors such as daylight, artwork, colours, organic shapes and access to nature are central to the salutogenic approach, as well as designs that make it easy for people to find their way around, Dilani says.

The Akershus University Hospital was designed to replace an older, much smaller hospital, and to serve the population of some 500 000 people living in and around Oslo.

It is efficient in terms of energy and staff time. About 85% of the hospital's heating and more than 40% of the total energy consumption is generated from a renewable source; geo-thermal energy. Windows in the wards can be opened, allowing for natural ventilation, in addition to air-conditioning. Distances that patients and staff travel within the hospital are short, giving the staff more time to attend to patients.

“The Akershus feels spacious and bright even on a grey Nordic day,” says Marie Sleveland, visitor coordinator and former head nurse, pointing out its many windows and 200m glass-roofed promenade. 

“The architects CF Møller sought to bring as much light into the hospital buildings as possible,” Sleveland says.

Architect Christian Dahle, one of the partners at CF Møller, refers to the style of the Akershus University Hospital as “inside out and outside in” because he and his colleagues used natural materials, such as wood, to reflect the surrounding fields and woodland landscape.

“Abundant light, fresh air, the use of natural materials inside and the ability to see nature outside, all these elements contribute to creating a healing environment in the Akershus hospital,” says Dahle.

In the glass-roofed promenade, there is a learning centre that provides courses for patients on how to manage diabetes, obesity and asthma. There are also shops and services, including a florist and a hairdresser that also sells wigs. “The hospital is a bit like a town with squares and meeting points,” says Dahle.

The views from the hospital windows onto the surrounding parkland and the landscape are also an important element of the design that aims to contribute to users’ wellbeing, Dahle says.

For example, the neonatal intensive care unit is situated on the top floor of the building, so that the families can have a beautiful view of the nature outside, Sleveland says.

“When you give birth and something goes wrong, it can be very stressful. There are bedrooms and a shared living room for the parents, who may stay for days, even weeks,” she says.

“Abundant light, fresh air, the use of natural materials inside and the ability to see nature outside, all these elements contribute to creating a healing environment in the Akershus hospital.”

Christian Dahle

Dahle recalls how the team of architects worked closely with hospital service user groups to develop the design that reflected their needs and values: “Working with them on the concept gave the users a sense of ownership and the feeling that it’s their hospital.”

Dahle credits Sleveland for her close coordination of the team of architects, doctors and patient/family user groups who were involved in the planning and design.

“When you are less stressed, you can work better,” Dahle says. “We concentrated on avoiding unnecessary distractions and barriers, both physical and visual, by making the outer structure and the inner design as user-friendly, bright and beautiful as possible.”

**Figure:**
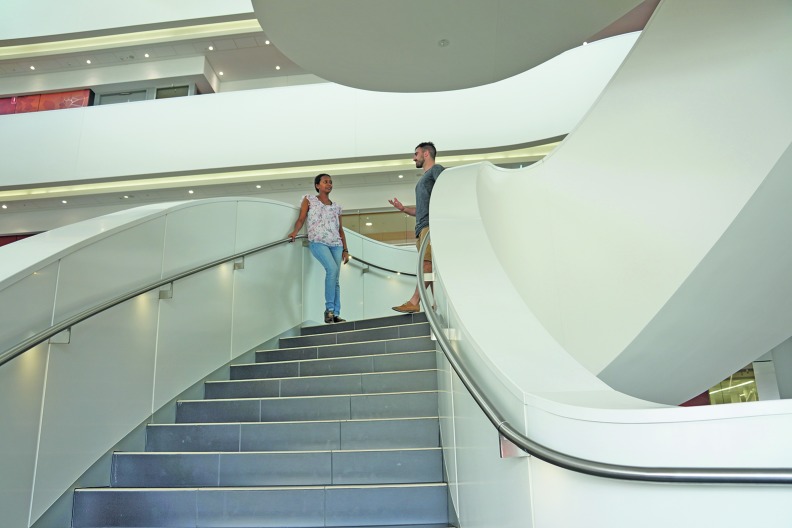
Melkam Kebede (left) meets a colleague on the stairs of the Charles Perkins Centre

The Charles Perkins Centre

**Figure:**
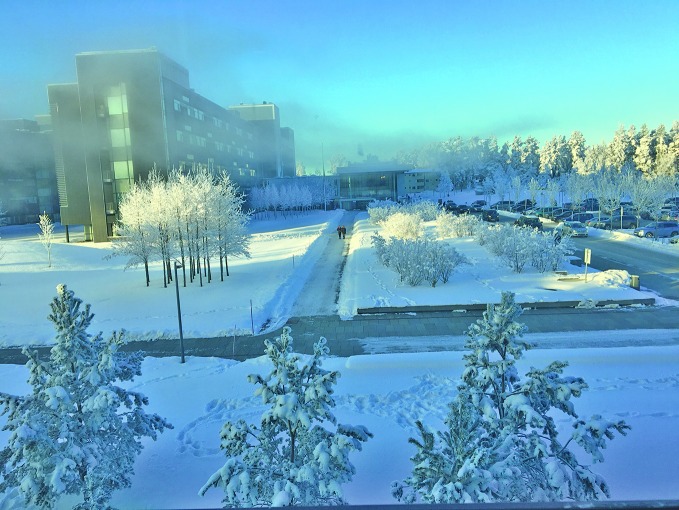
Akershus hospital with its surrounding parkland in winter

Akershus

